# Supplementary material for: Geometrical determinants of cerebral artery fenestration for cerebral infarction
Source: PeerJ. 2025 Jan 21;13:e18774. doi: 10.7717/peerj.18774 (PMC11758911; doi:10.7717/peerj.18774)
Supplement: Supplemental Information 1 [file peerj-13-18774-s001.docx]

Highlights

1. Study identifies key features of cerebral artery fenestration linked to stroke risk.

2. Significant differences in initiation and confluence angles observed in stroke cases.

3. Left-leaning fenestration type linked to higher risk of cerebral infarction.

4. Confluence angle and fenestration axes are independent stroke risk factors.

5. Early detection and management of fenestration crucial for stroke prevention.
